# Single-Molecule Fluorescence Defines a New Dye Chemistry

**DOI:** 10.1021/cbmi.5c00148

**Published:** 2025-11-04

**Authors:** Lujia Yang, Ying Zheng, Zhiwei Ye, Yi Xiao

**Affiliations:** † State Key Laboratory of Fine Chemicals, Frontiers Science Center for Smart Materials Oriented Chemical Engineering, 12399 Dalian University of Technology, Dalian 116024, China; ‡ College of Medical Laboratory, Dalian Medical University, Dalian 116044, China

**Keywords:** fluorescent dyes, super-resolution microscopy, single-molecule photophysics, single-molecule localization, brightness, switching kinetics, photostability, photochromism

## Abstract

Century-old fluorescent dyes have regained life in the wave of super-resolution microscopy. The achievement of single-molecule resolution transcends conventional ensemble averaging, imposing specific photophysical requirements on fluorescent dyes at a millisecond temporal resolution and ∼1 nm single-molecule spatial precision. This review aims to synthesize the fundamental principle governing single-molecule fluorescence in terms of single-molecule photon flux, switching kinetics, and the structure–function correlations. It emphasizes the role of structural modulation down to the atomic level for tuning single-molecule fluorescence characteristics to enhance imaging resolutions and proposes a taxonomy of landmark structural modifications for regulating single-molecule fluorescence. The analysis would provide guidelines for ongoing dye engineering efforts to develop next-generation dyes.

## Graphical abstract

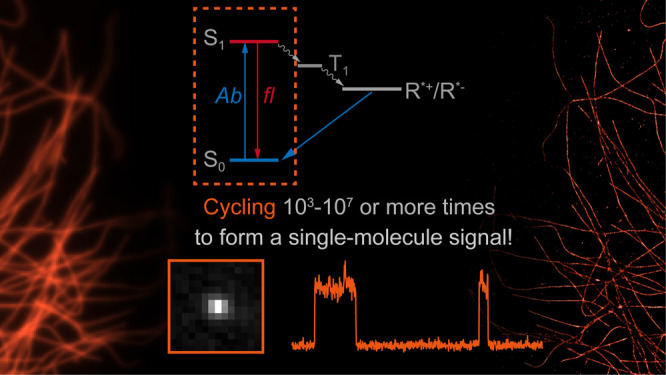

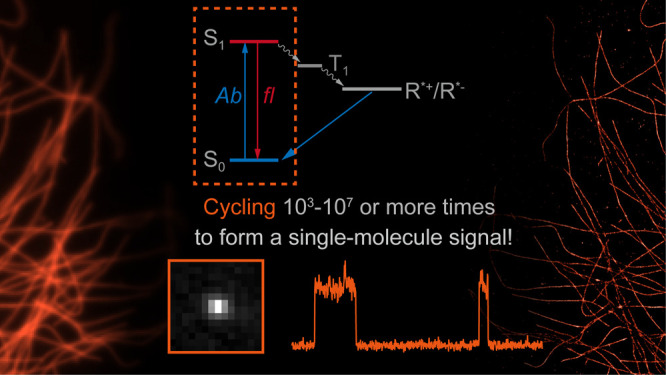

## Introduction

Fluorescent dyes have a long history. Since the first artificial synthetic dye, mauveine, in 1856, the scientific community has witnessed explosive growth in the structural tuning and molecular design of dyes.^1^ Prominent constructions have provided dyes with fluorescence quantum yield approaching ∼1,^2–4^ absorption coefficient surpassing 2.9 × 10^5^ M^–1^·cm^–1^ (Alexa Flour 750), huge Stokes shift up to >150 nm,^5,6^ or near-infrared emissions (>1000 nm).^7,8^ The accumulation of structure–fluorescence mechanisms has made rational molecular design essential for guiding dye studies today. Regarding these design rationalisms and “reaching-limit” properties, there seems to be no blank space left for future dye exploration. Does this imply a limitation for the dyes? The answer, however, is definitely not!

A new single-molecule chemical space has been opened up by the revolution in super-resolution microscopy for century-old dyes. Distinct from ensemble spectra and quantum behavior, single-molecule imaging in super-resolution imaging demonstrates unique single-molecule spatial and millisecond temporal resolution (Figure 1). This molecular resolution breakthrough shifts the field focus from ensemble averaging to individual molecules. The dyes in super-resolution imaging require precise on–off modulation of single-molecule fluorescence, arising from coupled photophysical transitions among ground (S_0_), singlet (S_1_), triplet (T_1_, intersystem crossing^9^) states, as well as photoreaction,^10^ photochromism,^11,12^ and other low-frequency pathways.^13–15^ The requirements for single-molecule fluorescence form a new chemical space over conventional dye studies.

**Figure 1:**
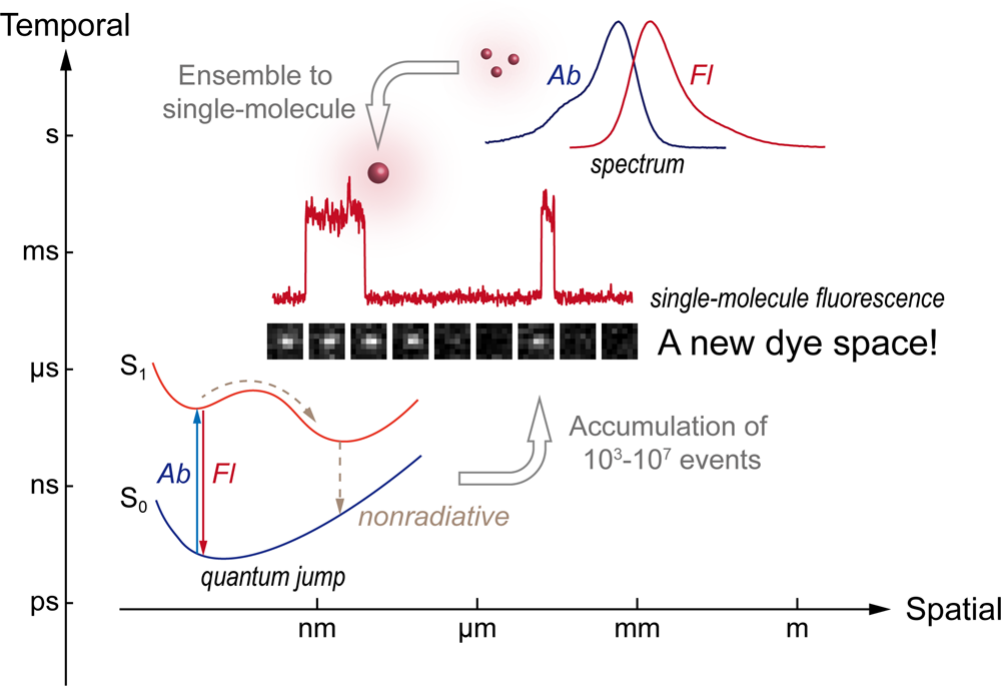
New single-molecule fluorescence chemical space for fluorescent dyes. Single-molecule fluorescence, trajectories, and spectra are reproduced from refs 16 and 17. Copyright 2005, Royal Society of Chemistry, and 2020 Dalian University of Technology.

Switching between the bright and dark forms the core of single-molecule fluorescence. Single-molecule resolution is obtained by identifying and precisely localizing the point spread function (PSF) from individual dye molecules. The PSF consists of 10^3^–10^5^ photons, corresponding to a greater number of S_1_–S_0_ fluorescence cycles for a single-dye molecule before it transitions to the dark state. Yet, the dark dwelling is required to isolate bright molecules in both spatial and temporal dimensions. Thus, even though the inhibition of transition probability to the dark state improves single-molecule brightness, it reduces the population of dark states, endangers individuality, and raises the concern of molecular overlap. Thus, careful molecular design is required to navigate this trade-off to attain a high population of molecules in dark states while maintaining the bright state for increasing the photon yield on each on-event.

Modulation of single-molecule fluorescence has been an emerging field in recent years. Although many excellent reviews have summarized the fluorescence structure relationships,^18–20^ they have mainly focused on ensemble averaging optimization, obscuring the heterogeneity of single molecules. It is necessary to comprehensively understand the structural relationship of single-molecule fluorescence to establish a molecular foundation for designing high-performance super-resolution probes.

Herein, through an in-depth analysis of recent key research findings and experimental data, we analyzed the structural insights to enhance single-molecule fluorescence, thereby aiming to promote the development and innovative applications of super-resolution imaging technology.

### Photophysics Requirements for Dyes in Super-Resolution Microscopies

The mechanisms of super-resolution microscopies are key to understanding the photophysical requirements of dyes. Various super-resolution microscopies have been developed and divided into three major categories on the basis of their working principles (Figure 2). The first category, single-molecule localization microscopy (SMLM), including photoactivated localization microscopy (PALM),^21^ stochastic optical reconstruction microscopy (STORM),^22^ and ground-state depletion imaging microscopy (GSDIM),^23^ relies on temporally separating the fluorescence of individual emitters and fitting their PSFs with nanometer precision. Other fluctuation-based methods, such as super-resolution optical fluctuation imaging (SOFI),^24^ do not require the isolation of a single emitter, but extract high-order statistical correlations from temporal fluorescence fluctuations to achieve resolution enhancement. The second category includes structured illumination microscopy (SIM)^25^ and its advanced variants such as Hessian structured illumination microscopy (Hessian SIM)^26^ and saturated structured illumination microscopy.^27^ These techniques rely on patterned illumination to extract high-frequency information to achieve a resolution enhancement. The third category includes stimulated emission depletion (STED) microscopy,^28^ which constructs a doughnut-shaped depletion beam alongside an excitation beam to achieve subdiffraction localization. The stimulated dyes under the doughnut-shaped beam did not emit fluorescence, except when they were located at the center of the beam. Thus, a super-resolution is obtained by scanning the sample with both the doughnut stimulation beam and the excitation beam. Recently, minimal photon flux (MINFLUX) microscopy^29^ has been developed, which combines the principles of STED and single-molecule localization microscopy. This unique approach achieves prominent subnanometer resolution (<1 nm) by scanning the small volume of a spatiotemporally isolated dye molecule (like those obtained in SMLM) with doughnut beams. In contrast to the stimulation process in STED imaging, the dye molecule is excited during the beam scanning until it is located at the center of the doughnut. Thus, the elimination of stimulation significantly reduces the photon budget, and this method is named minimum photo flux.

**Figure 2:**
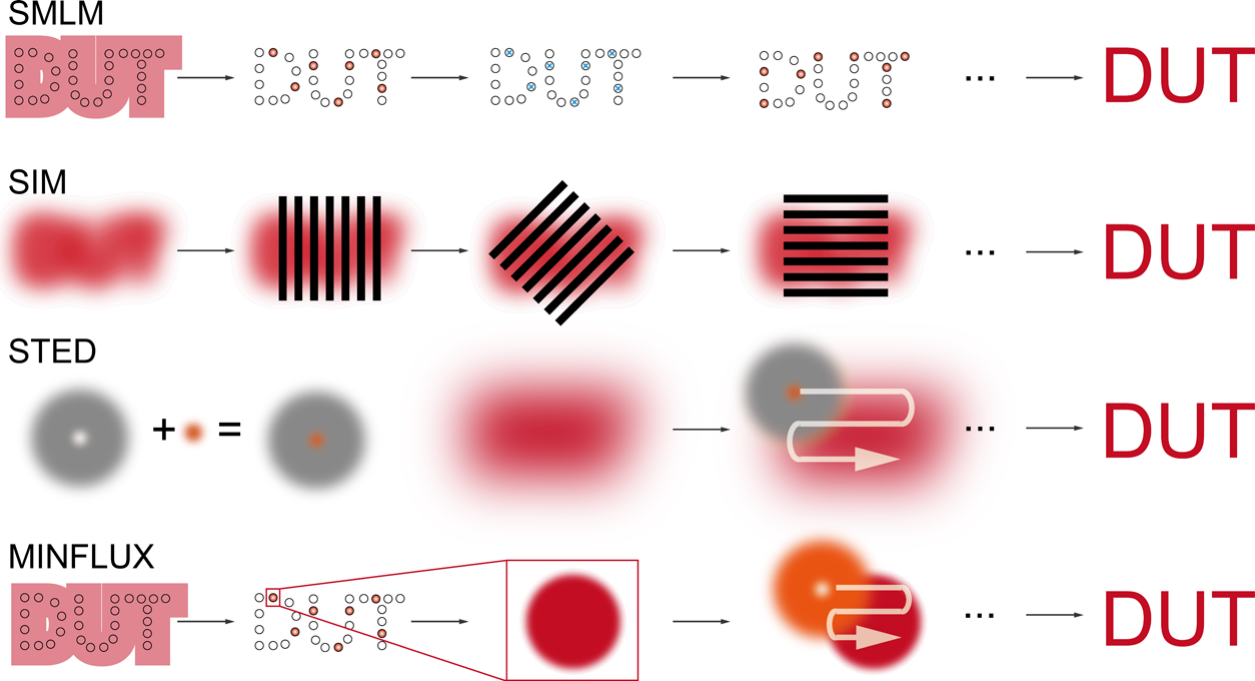
Principle of the imaging technique.

Each of these approaches imposes distinct photophysical requirements on fluorescent dyes. First, SMLM demands controllable photoswitching and high photon output from a single-dye molecule, which is the strict single-molecule fluorescence photophysics discussed subsequently. On the other hand, SIM microscopy with a ∼100 nm resolution tolerates conventional bright dyes. Although some advanced SIM techniques like Hessian and saturated SIM might impose high photo budgets to achieve enhanced resolution, their dye photophysics do not decrease to a single-molecule level. Moreover, the dye photophysics for STED and MINFLUX are different, even though both utilize a doughnut-shaped beam. STED requires extreme photostable stimulated emission for dyes, which is distinct from the fluorescence photostability of a dye with bare singlet–triplet-state transition involvement. In MINFLUX, the use of a doughnut-shaped beam is reversed. Once a single emissive molecule is identified, the doughnut beam scans over a small region that it presents to find the true location of the molecule. Thus, it shares a temporal separation requirement similar to that of SMLM, and theoretically the dwelling of a dye bright state should be confined to match the duration of the beam scanning.

The deciding roles of dye photophysics across many super-resolution imaging methods provide the rationale for the subsequent discussion of structural modification strategies. To keep the focus on the single-molecule fluorescence, this review primarily discusses dyes used in SMLM and MINFLUX; for dyes employed in other techniques that do not operate at single-molecule resolution, readers are referred to other prominent reviews.^30,31^ To emphasize the structural evolution of dyes, the applications of these dyes are only briefly mentioned, with their specific super-resolution imaging types and labeling approaches labeled in the figures.

### Single-Molecule Fluorescence

Before analyzing the structural origin of single-molecule fluorescence, it is essential to first reach a common framework for versatile single-molecule fluorescence propensities across research. Figure 3 exhibits a simple two-state single-molecule trajectory, which is extracted from the pixels representing the same molecule on the acquisition frame stacks. Every point on the trajectory corresponds to the brightness of a single-molecule signal at a specified time interval defined by the frame stack. The most prominent characteristic is the fluctuation of single-molecule fluorescence, which forms the core identity over ensemble averaging. The small fluctuation is a consequence of camera noise (readout noise, randomness from the electron multiplier), molecular rotations, and diffusions. Fluorescence correlation spectroscopy would provide the intrinsic lifetime for these processes.^32^ For this review, the focus is narrowed to the major transition between dark and bright states, and these small fluctuations are omitted.

**Figure 3:**
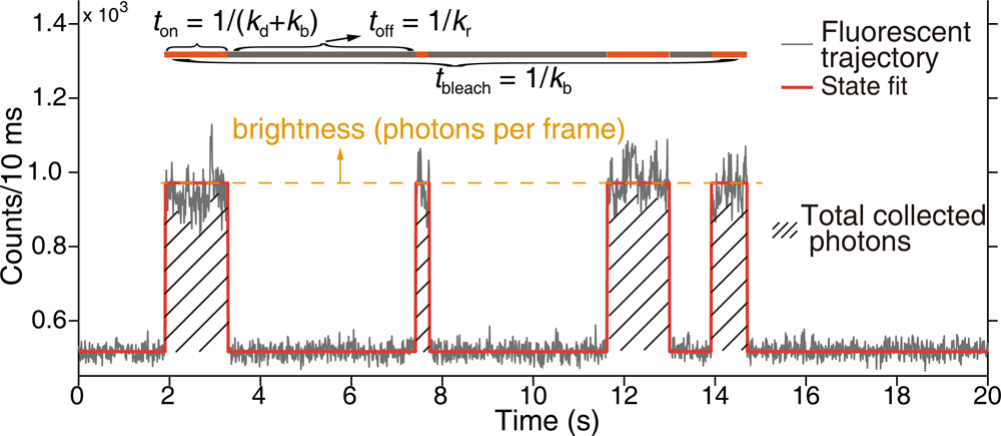
Single-molecule fluorescence and abstracted characteristics. Reproduced from refs 17. Copyright 2020, Dalian University of Technology.

Single-molecule brightness and switching kinetics are the basic building blocks of single-molecule fluorescence. The former defines the signal-to-noise ratio of a single-molecule signal, whereas the latter defines the dwelling behavior of a dark-bright transform. The quantification of single-molecule brightness is achieved through many parameters, including but not limited to the emissive rate (*r*_em_), photon flux, photons per frame, photons per switching event, total collected photons before photobleach (*N*_photons_), etc. The emissive rate (also called photon flux and photons per frame) is utilized to accurately describe the signal acquired, reflecting the photon burst from a single-dye molecule. The total number of photons collected before photobleaching was not included, as this parameter could be derived from the product of the single-molecule brightness and the total dwell time of on states (*N*_photons_ = *r*_em_ × ∑*t*_on_).

Switching kinetics are generally abstracted to three rates: the dark-bright transform rate *k*_r_, the bright-dark transform rate *k*_d_, and the bleach rate *k*_b_. These rates are facilely obtained as the rate *k* of a Poisson process (*p*(*t*) = *k*e^–*kt*^), and transformed to dwell times by inversing (*t*_off_ = 1/*k*_r_, *t*_on_ = 1/(*k*_d_ + *k*_b_), and *t*_bleach_ = 1/*k*_b_). Note that the rates and times do not rigidly restrict the switching behaviors of a single molecule, and it is not possible to directly link the quantified rates or dwell times to the switching behaviors of a single-dye molecule. Yet, the switching events of the dye molecules are counted and fitted to Poisson processes with these rates or times, as expected. Through quantification, the switching kinetics (*t*_on_, *t*_off_, *t*_bleach_) alongside the single-molecule brightness (emissive rate and photons per switching event), it is possible to generalize the single-molecule fluorescence of a dye.

To simplify the switching kinetics, Zhuang et al. proposed a duty cycle (the total dwell time of a dye molecule in its bright states versus the total imaging time) to estimate the fluorescence sparsity of a dye molecule and exemplify its super-resolution imaging potential.^33^ However, the photobleaching process is omitted in the reporting of duty cycles since an imaging-enhancing buffer with a deoxygenated condition was involved in that study. Yet, studies in the dye field favor unclosed single-molecule photophysics without any additives in which photobleaching necessarily occurs. In addition, the duty cycle is often mistakenly developed, overlooking the single-molecule preconditions. Caution should be taken to report this value, as it is probable to report a similar ratio of dwell-on times for multiemitter conditions. Thus, a new property involving photobleaching is required for a better comparison of the switching distinctiveness between new dyes. We suggest a recruiting yield (Φ_r_ = *k*_r_/(*k*_r_ *+ k*_d_ + *k*_b_)) and its negative logarithm (*p*Φ_r_ = −log_10_(Φ_r_)) for abstracting the switching efficiency (ε_switch_ = *p*Φ_r_) in one term. A higher ε_switch_ value indicates that more molecules exist in the dark state during imaging. Thus, dyes with high ε_switch_ values are preferred for densely labeled imaging.

Although the single-molecule fluorescence of a dye is well quantified by the above parameters, the defined system has a major constraint on laser dependency. Most of these parameters are not laser-free, which highly restricts their reliability. For instance, the single-molecule brightness is enhanced if a high-power laser is utilized, except when it reaches saturation. Nevertheless, most of the studies were performed under 500–2000 mW/cm^2^, a relatively high laser power, making the comparison between them practical. In addition, it is strongly encouraged for researchers in the field to search for laser-free single-molecule parameters (like saturated emissive rate and the slope of the light power versus emissive rate plot, which might be a molecular characteristic independent of the laser). Next, single-molecule brightness and switching kinetics would be separately analyzed according to their molecular structures.

### Single-Molecule Brightness

Single-molecule brightness is not merely the product of the molecular absorption coefficient (ε, also identified as the cross section) and fluorescence quantum yield (Φ), but also depends on the dwelling photophysics of bright forms, which corresponds to a large number of cycles between the S_1_ and S_0_ states. Notably, the former product (ε × Φ) has been accepted as the general brightness of a dye with many recent landmark contributions. Thus, the discussion of structural insights from single-molecule brightness naturally starts from this term and goes deeper into the molecular modulation achieving enhanced cycling persistency (Figure 4). It should be noted that to explicitly understand the molecular structure toward single-molecule brightness, not all works on fluorescent dyes are included.

**Figure 4:**
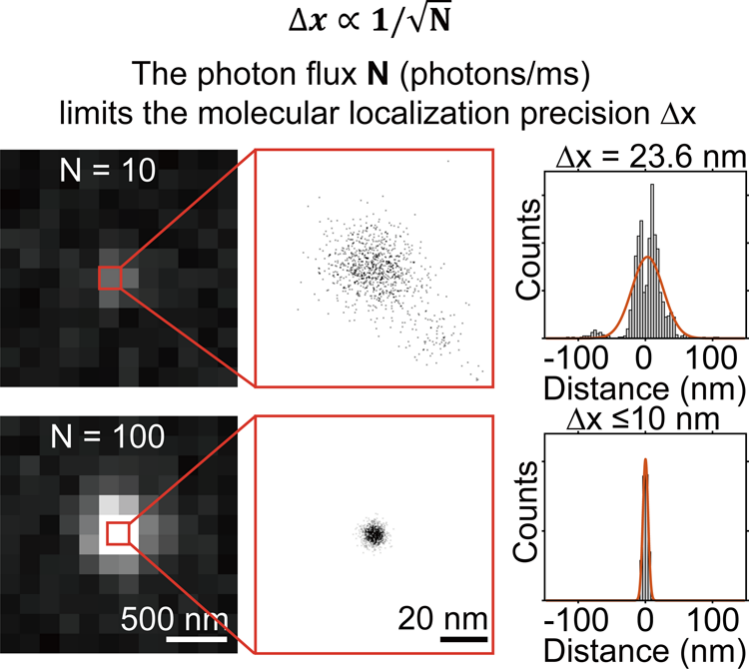
Single-molecule brightness defines the localization precision. Reproduced from ref 17. Copyright 2020, Dalian University of Technology.

The fluorescence quantum yield indicates the possibility that a molecule emits a photon in its excited state. Thus, the inhibition of nonradiative decay constitutes a basic approach to improve brightness. The most well-known nonradiative pathway might be twisted intramolecular charge transfer (TICT). The twisting of the amine donor groups breaks the conjugation plane and results in energy dissipation of the excited states (Figure 5). Modulation of steric and inductive electron effects constitutes two general strategies for the inhibition of TICT formation. In the former strategy, steric hindrance restricts the rotation of the amine group during excitation and thus improves the possibility of fluorescence pathways. Bridged amines with rigid structures and bulky sizes were first introduced to enhance the fluorescence of rhodamines by Foley in 2007.^34^ The obtained 7-azabicyclo[2.2.1]­heptane rhodamine presented a 0.95 quantum yield. Later, highly strained azetidine amine was introduced by Lavis in a series of landmark works.^3,35,36^ Their developed Janelia Flour series dyes exhibited Φ ≥ 0.88 in aqueous solution. JF_549_ (Figure 5, **1**) exhibited a high photon emission rate of 19 photons/ms. We further expanded the modulation armories through electron induction modulation.^2^ Quaternary ammonium piperazine was deployed to rhodamines, naphthalene, and NBD push–pull fluorophores. The significantly enlarged electron-withdrawing functionality of ammonium units creates a high energy barrier, inhibiting the formation of TICT states. The developed *N*,*N*-dimethylpiperazine-substituted rhodamine (Figure 5, **2**) exhibits a 0.92 quantum yield in aqueous solution and a single-molecule emissive rate of ∼109 photons/ms. Later, the Guo group has introduced a similar electron-withdrawing strategy with sulfone groups.^37^ Recently, the Chen group has combined the steric and electron-withdrawing effect into one, demonstrating a bridged bicycle-strengthened fluorophore (Figure 5, **3**) with a quantum yield up to a record high of 0.98.^4^

**Figure 5:**
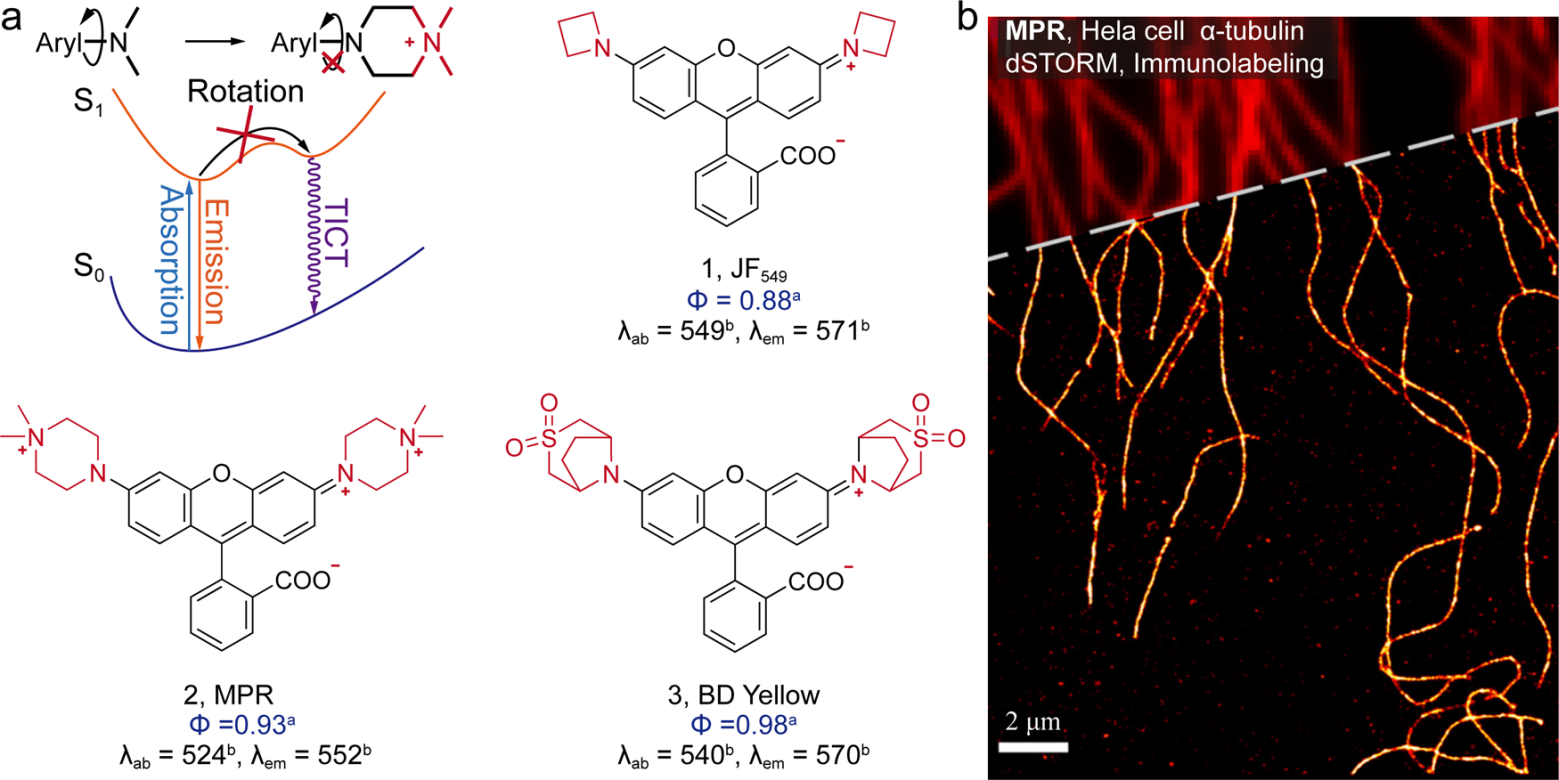
(a) Molecular design to inhibit the twisted intramolecular charge transfer. ^a^Quantum yield was measured in a water solution. ^b^Peak wavelength in nm (the same superscript following). (b) Super-resolution image of microtubules immunolabeled with a primary antibody against α-tubulin and MPR-IgG. A conventional image is overlaid on the top. Reproduced from ref 2. Copyright 2019, American Chemical Society.

Fluorinated substitutions also form a key strategy for improving the quantum yield (Figure 6). This atom exchange could induce electron-withdrawing functionality, rigidify the scaffold, and reduce internal conversion. The Hell group has constructed libraries of fluorinated silicon rhodamines and phosphorylated oxazines (Figure 6, 4) with high quantum yields (0.70) and bathochromic spectral shifts.^23^ The Yuan group has implemented the trifluoromethyl design in their large Stokes shift dyes YL578 (Figure 6, **5**), and successfully enhanced the quantum yield from 0.31 to 0.74^5^. The Hell group has developed trifluoromethyl-substituted coumarins (Figure 6, **6**) with a quantum yield of up to 0.8 in ethanolic solution.^38^ The quantum yield of diarylethene could also be tuned by the electron induction effect on substitutions at the 6,6′-positions of the benzothiophene moiety. The Irie group has developed fluorinated diarylethene with phenyl or thiophene ring substitutions to suppress nonradiative decay processes (Φ = 0.88 in 1,4-dioxane).^12^

**Figure 6:**
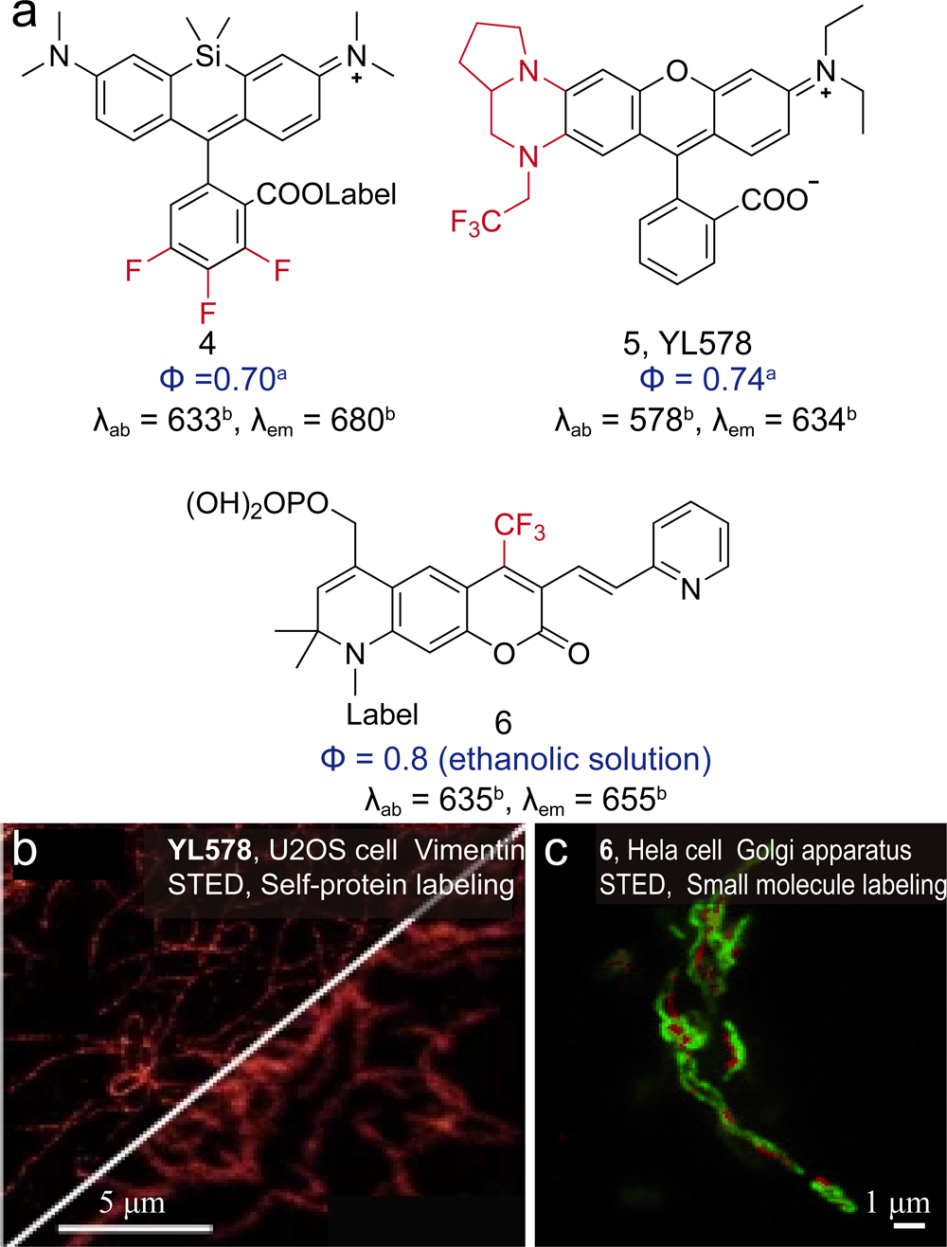
(a) Fluorinated dyes with improved brightness. (b) Confocal and STED images of live U2OS cells stably expressing vimentin-HaloTag labeled with YL578. Reproduced from ref 5. Copyright 2022, Springer Nature. (c) STED two-color imaging of the Golgi apparatus. Reproduced from ref 38. Copyright, 2013 Wiley-VCH.

The past has witnessed an inspiring increase in the quantum yield through structural modulation. Yet, it does not fully depict the picture of single-molecule brightness. It is possible to have fluorophores with a quantum yield of ≥ 0.5, but they may fail to present an effective single-molecule signal. The secret lies in the “nonradiative” pathways, which might form temporary (μs–ms level) nonfluorescence states (triplet states, configuration transforms like *cis*–*trans* isomerism) to inhibit consistent emission from a single fluorophore. The ring rigidification of Cy3 was already established in 2000.^39^ It was not until recently that Martin et al. found the routes and synthesized *trans*-conformation-rigidified Cy5B (Figure 7, **7**).^40,41^ This new dye demonstrated a significantly enhanced quantum yield (Φ = 0.69) as well as persistent photo flux, attributed to the elimination of microsecond-scale dark states. We have developed an acid strategy to decrease the possibilities of temporary dark states for rhodamine spirolactam. A carboxylic acid group is placed proximate to the spiro ring (Figure 7, **8**).^42^ Upon proton or photoactivation, the acquired zwitterionic bright state is stabilized by probable intramolecular hydrogen bonding. Thus, the acquired amino acid rhodamines exhibited an extended lifetime of the bright state and a 1.2-fold enhancement in single-molecule brightness (36 photons/ms).

**Figure 7:**
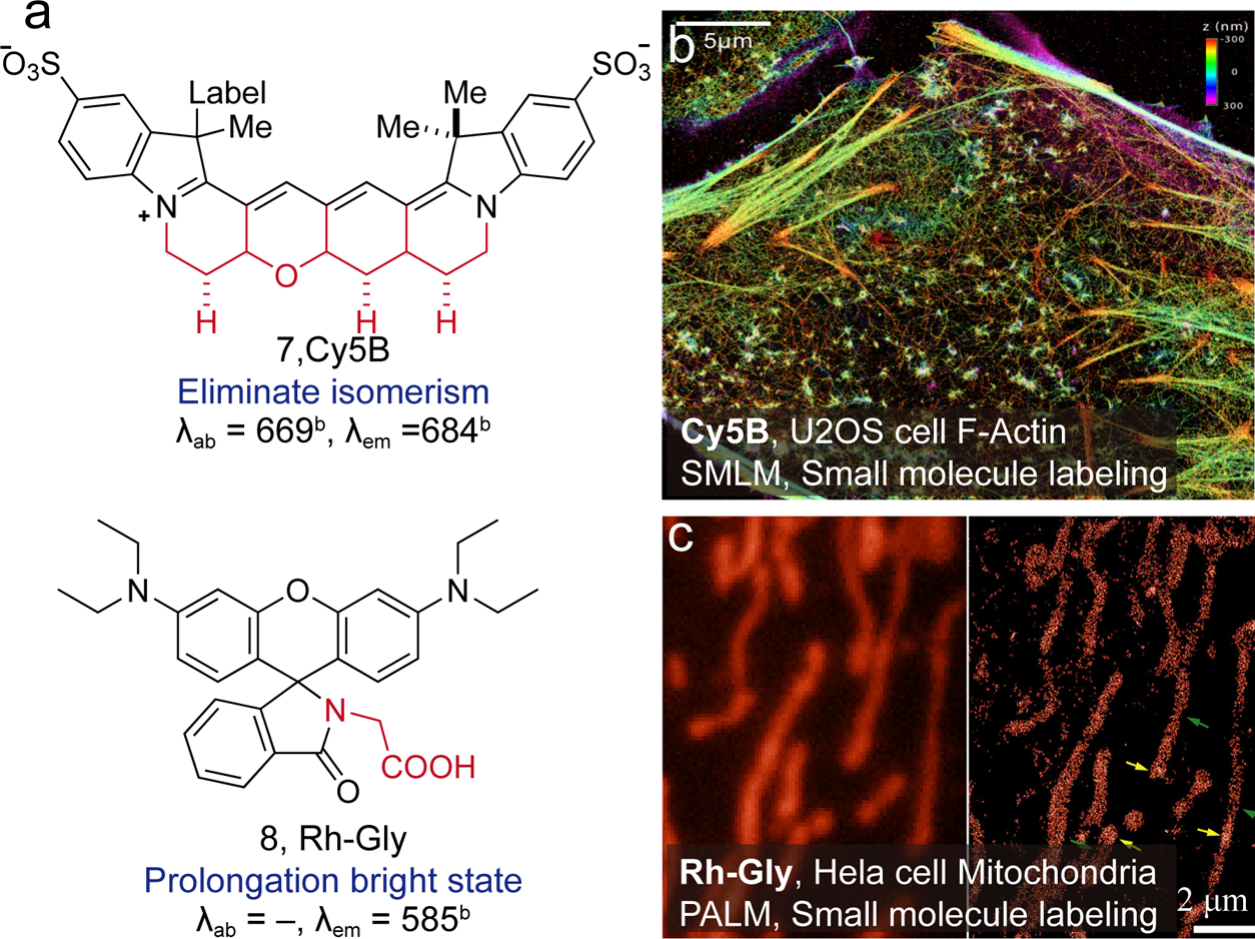
(a) Stabilization construction for improving single-molecule brightness. (b) High-quality 3D TRABI-BP SMLM image of F-actin in U2OS cells. Reproduced from ref 40. Copyright 2017, American Chemical Society. (c) Super-resolution imaging of the mitochondria-enriched regions in live HeLa cells stained with Rh-Gly. Reproduced from ref 42. Copyright 2019, American Chemical Society.

Another major breakthrough was the development of self-healing dyes. Through the spatial proximity introduction of triplet quencher units, the suspension of emissions caused by intersystem crossing was largely inhibited (Figure 8). Blanchard group^43^ first suggested and largely implemented this strategy for various commercial dyes, which dramatically prolonged their dwelling in the bright state. They further investigated the mechanism behind this prolongation through the position study of the typical triplet quenchers (1,3,5,7-cyclooctatetraene, COT; trolox; nitrobenzyl alcohol, NBA).^44,45^ It was inferred that a collision between the excited state and the quencher depletes the triplet states. Subsequently, Li et al. designed a self-healing near-infrared fluorescent dye, NPA-Cy5.5,^46^ by covalently linking the triplet-state quencher 4-nitro-3-phenyl-l-alanine (NPA) to Cy5.5 dye and further conjugating it to a zwitterionic polypeptide polymer. Another interesting enhancing unit is tris-*N*-nitrilotriacetic acid (NTA), which is used to bind proteins; whereas the fluorophore bound to the NTA structure exhibits persistent single-molecule emission. Cosa et al. and Cordes, Sauer et al. individually developed rigid linkers (rigid polyproline-II (PPII) helix and proline helix linkers) to enhance the single-molecule performance,^47,48^ both of which enhanced the quantum yield and single-molecule brightness. The rigid linker exhibited a bifunction that inhibited quenching and enhanced stability. Single-molecule experiments demonstrated up to a 25-fold increase in the photon output, with a photon flux of ∼6 photons/ms and markedly prolonged photobleaching lifetimes. However, the prominent issue is the unignorable reduction of emissive rates by the coincident electron transition from the excited S1 state to the quencher, which significantly lowers the signal-to-noise ratio of their single-molecule signals. Meanwhile, the “healing” stability also made a barrier for further photoswitching, although the Cordes group has suggested the addition of enhancing agents for mitigation.^49^

**Figure 8:**
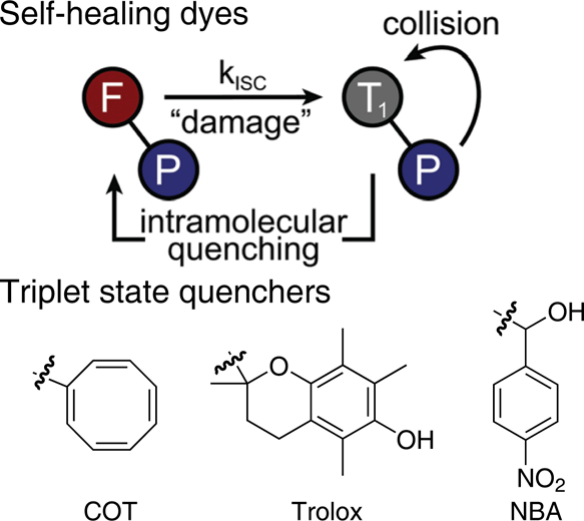
Mechanism of self-healing and typical triplet-state quenchers. Reproduced from ref 50. Copyright 2020, American Chemical Society.

From the molecular structures to the proximate quencher units, it is inferred that the surrounding environment further heavily impacts the single-molecule brightness of a dye. The pH is a regulator of single-molecule brightness. We have identified an increase of single-molecule brightness from 98 to 139 photons/ms in acidified cellular lysosomes.^42^ Xu group has investigated the single-molecule brightness at various pH values, indicating an increment of the emissive rate at high proton concentration (from 42 photons/ms, pH 7.0 to 45 photons/ms, pH 4.0).^51^ Besides the protonation and hydrogen-bonding effect, the London dispersion force and the environmental spatial conformation influence the emission rate significantly. Klymchenko et al. have chemically modified the classical plasma membrane probe FM1–43 with a sulfonate group,^52^ and a new dye with a neutral zwitterionic structure enhanced its brightness (Φ = 0.26 in liposomes) upon binding to the plasma membrane surface. Fürstenberg et al. have developed a host–guest system for the rigidification of the dye environment for imaging.^53^ They utilized the intraspace of cucurbituril (CB) to encapsulate the dye molecules. Upon binding to CB, the dyes exhibited a ∼40% increase in the fluorescence quantum yield and a 32% improvement in the single-molecule photon flux of ATTO655.

Beyond the scope of structural and environmental strategies, reverse thinking of cleaning the background would further “enhance” the single-molecule brightness. We have introduced o-phenylenediamine to inhibit the ring-opening of spirolactam through Coulombic repulsion (Figure 9, **9**), obtaining a single-molecule brightness of 112 photons/ms.^51^ Butkevich, Hell group replaced the carbonyl with an imine moiety for their byproduct-free photoactivatable 1-vinylxanthone (PAX) dyes (Figure 9, **10**),^54^ which significantly reduced background fluorescence under preactivation.

**Figure 9:**
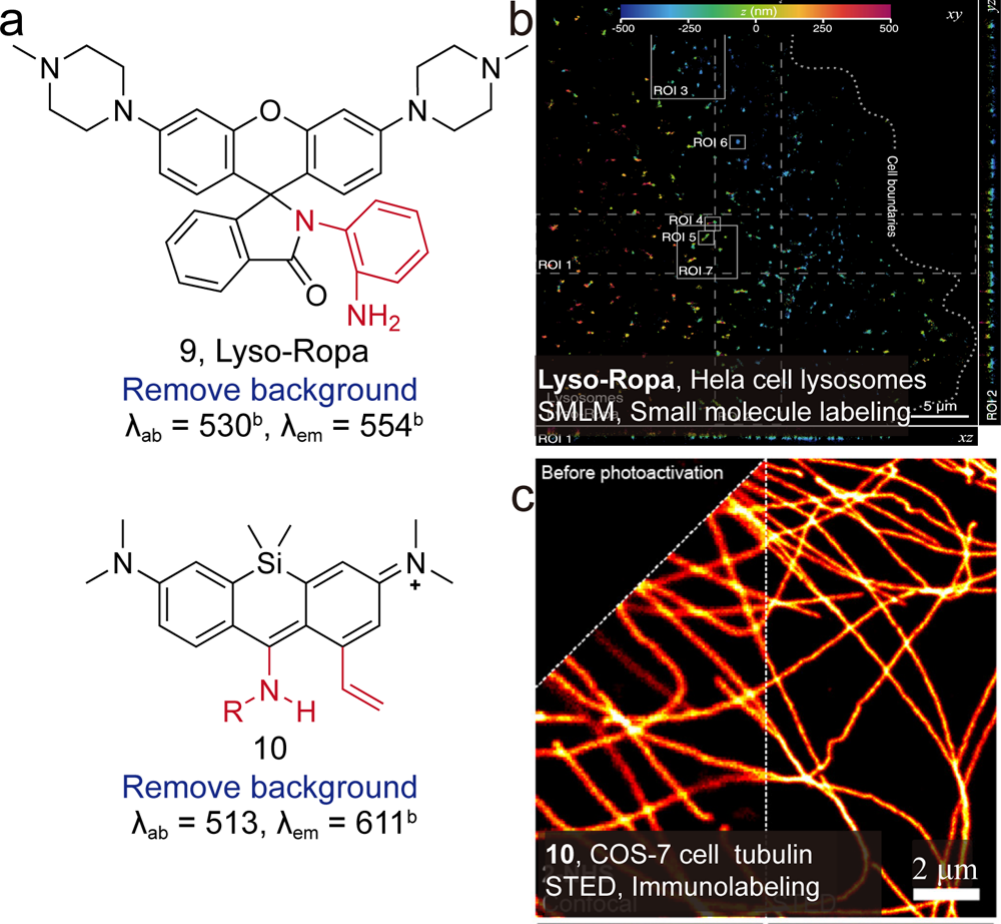
(a) Dyes engineered to eliminate background for enhancing brightness. (b) Multidimensional SMLM imaging of lysosomes in live HeLa cells by no-wash Lyso-Ropa staining. Reproduced from ref 51. Copyright 2022, American Chemical Society. (c) Confocal and STED images of tubulin filaments in fixed COS-7 cells labeled by indirect immunofluorescence with secondary antibodies tagged with **10**. Reproduced from ref 54. Copyright 2023, American Chemical Society.

Single-molecule brightness is rooted in the molecular structure and the surrounding environment. Although the fluorescence quantum yield provides a measure of brightness, it does not present a full picture of single-molecule brightness. The emission durability and burst (emissive rate) should be further investigated by considering the structural insights of the dye. Figure 10 displays the ladder of emissive rates from some dyes reported for reference. Yet, the drawback of laser dependency of the emissive rate and photon collection of a single bright state might be a barrier for future analysis; the saturated emissive rate is suggested to measure the real brightness limit of a single-dye molecule.

**Figure 10:**
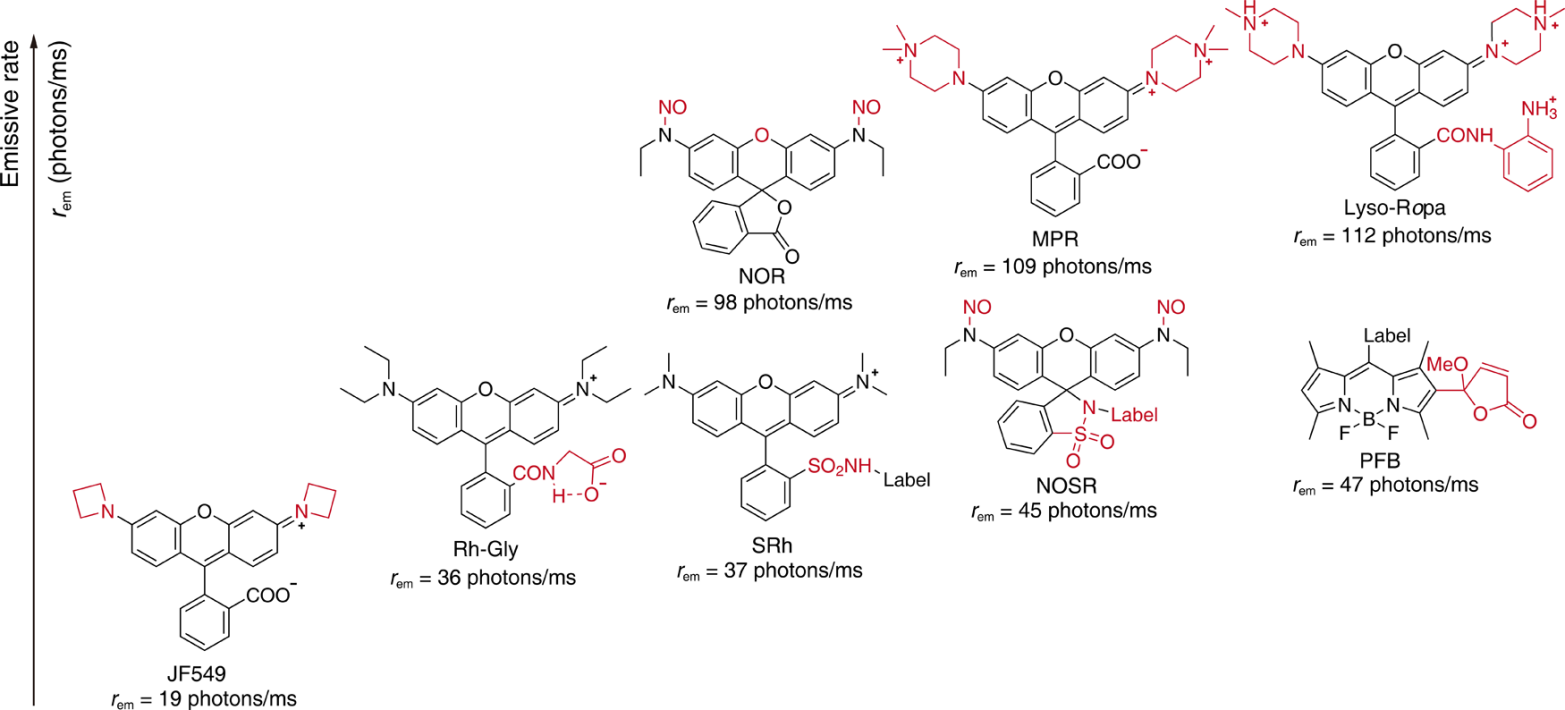
Ladder of emissive rates for some reported dyes.

### Switching Kinetics

The switching kinetics form the other core of single-molecule fluorescence. The observation of the occasional disappearance of single-molecule fluorescence dates back to the early 20th century. The dark states are attributed to the stochastic transition to triplet states until the breakthrough in switching engineering (Figure 11). Through the implementation of a pair of Cy3–Cy5 dye systems, Zhuang group^55^ demonstrated the control of the dark–bright states of Cy5 dyes. This inspiring progression, as well as the development of photoactivatable fluorescent proteins, finally triggered the first single-molecule localization microscopies (STORM,^22^ PALM,^21^ and fPALM^56^). It is apparent that switching forms a key building block for molecular localization imaging. Thus, how do we control the seemingly stochastic nature of a dye molecule?

**Figure 11:**
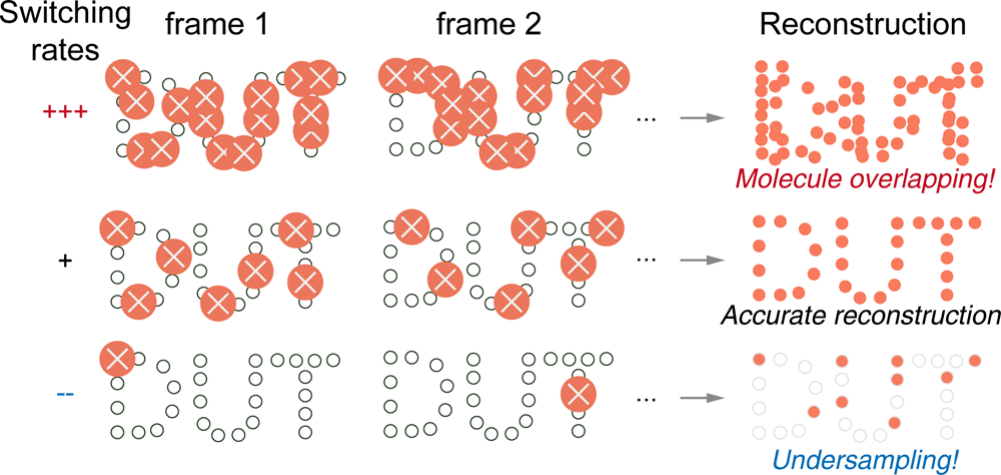
Switching kinetics define the reconstruction accuracy.

To control switching, the underlying mechanism must first be investigated. The first question is: what is a dark state? The investigations suggest that *trans*–*cis* isomerization^11^ and thiol adduct^57^ correspond to the dark states of the Cy5 dye (Figure 12). More generalizable, the conformation transformation, photoreaction, and oxidation-reduction processes might form basic building blocks for switching. It is noticeable that these processes are not isolated but cross-correlated with each other. For instance, *trans*–*cis* isomerism would enhance nucleophilic photoaddition to the thiol adduct, which might present a dwelling dark time of >1 s. Recently, the intrapathway for a single Cy5 dye in the switching buffer has attracted much interest. Harriman et al. reported that under conditions without strong reducing agents, AF-647 undergoes photoisomerization to form at least two long-lived dark-state isomers.^58^ Cosa group found that thiols induce the formation of triplet exciton pairs via photoinduced electron transfer.^57^ These exciton pairs undergo competitive pathways of either reverse electron transfer or radical recombination, resulting in photostabilization or the formation of nonemissive states, respectively. Inspired by the thiol-addition strategy in the imaging-enhancing buffer, Zhuang et al. developed a universal reductive blinking activation strategy by introducing triphenylphosphine (TPP) into the imaging system of cyanine dyes, such as Cy5.^59^ This approach relies on the formation of a reversible, nonfluorescent adduct between TPP and the excited-state dye, which modulates the transition rate between the on and off states, thereby tuning the switching kinetics. By modulation of the pH and laser power, the dark-state lifetime of the Cy5-thiol adduct can be controlled, thereby optimizing the switching kinetics. A thorough discussion of the photophysics of cyanine dyes has been provided by Levitus and Ranjit.^60^ Thus, these details are omitted here, and readers might find it helpful to directly read the excellent review. Nevertheless, the complexity of the pathways for switching suggests difficulty in tuning switching.

**Figure 12:**
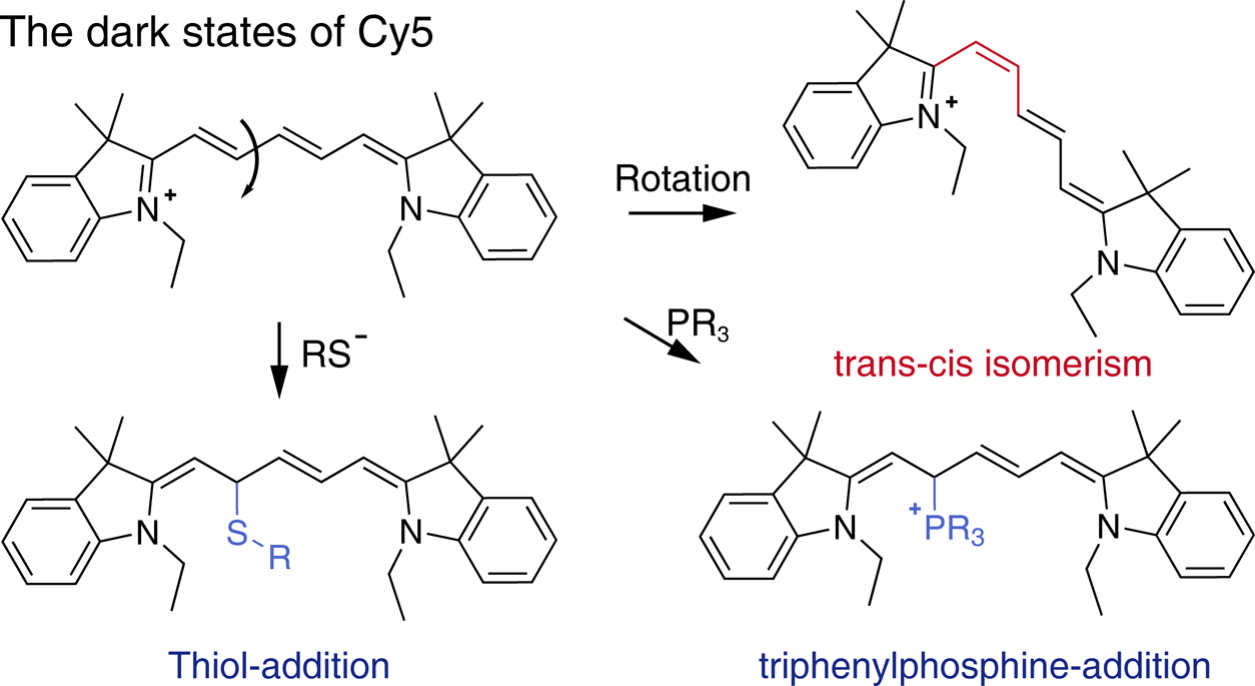
Molecular structures of the dark states of Cy5.

Despite this hardship, researchers have continually modulated switching behaviors through molecular structures. Xu et al. significantly optimized the photoswitching behavior of rhodamine dyes by replacing the central benzene ring with a permanently positively charged 1,3-disubstituted imidazolium moiety (Figure 13, **11**).^61^ This structural modification enhanced the dye’s electron-accepting ability and stabilized the radical dark state through resonance effects. As a result, the imidazolium-substituted dye exhibited rapid and reversible switching kinetics in a deoxygenated buffer along with markedly increased single-molecule photon output and long-term photoswitching stability. Despite the commonly adopted rhodamines and cyanines, Müllen and Peneva et al. developed a guanidinium-functionalized perylene monoimide (Figure 13, **12**) and naphthalene monoimide dyes.^62^ The introduction of unsymmetrical units granted electron separation for generating a possible electronic recombinant dark state. The new dye showed blinking behavior suitable for super-resolution localization and super-resolution imaging. Cadby and Jones et al. synthesized a series of 4-nitrobenzochalcogenadiazole dyes.^63^ Sulfur substitution enhanced the molecular aromaticity and reduced the triplet-state stability, thereby shortening the dark-state dwell time and increasing the quantum yield. In contrast, selenium substitution led to red-shifted absorption and emission spectra but resulted in lower quantum yields and extinction coefficients, indicating that the heavy atom effect promoted intersystem crossing and increased the likelihood of dark-state formation. It is worth mentioning that an expansion of the dye palette is required, as very few classes of dye scaffolds (mostly rhodamines, cyanines) have been developed for molecular localization super-resolution imaging. The molecular structural transformation forms fundamental pathways for tuning dye molecule photophysics.

**Figure 13:**
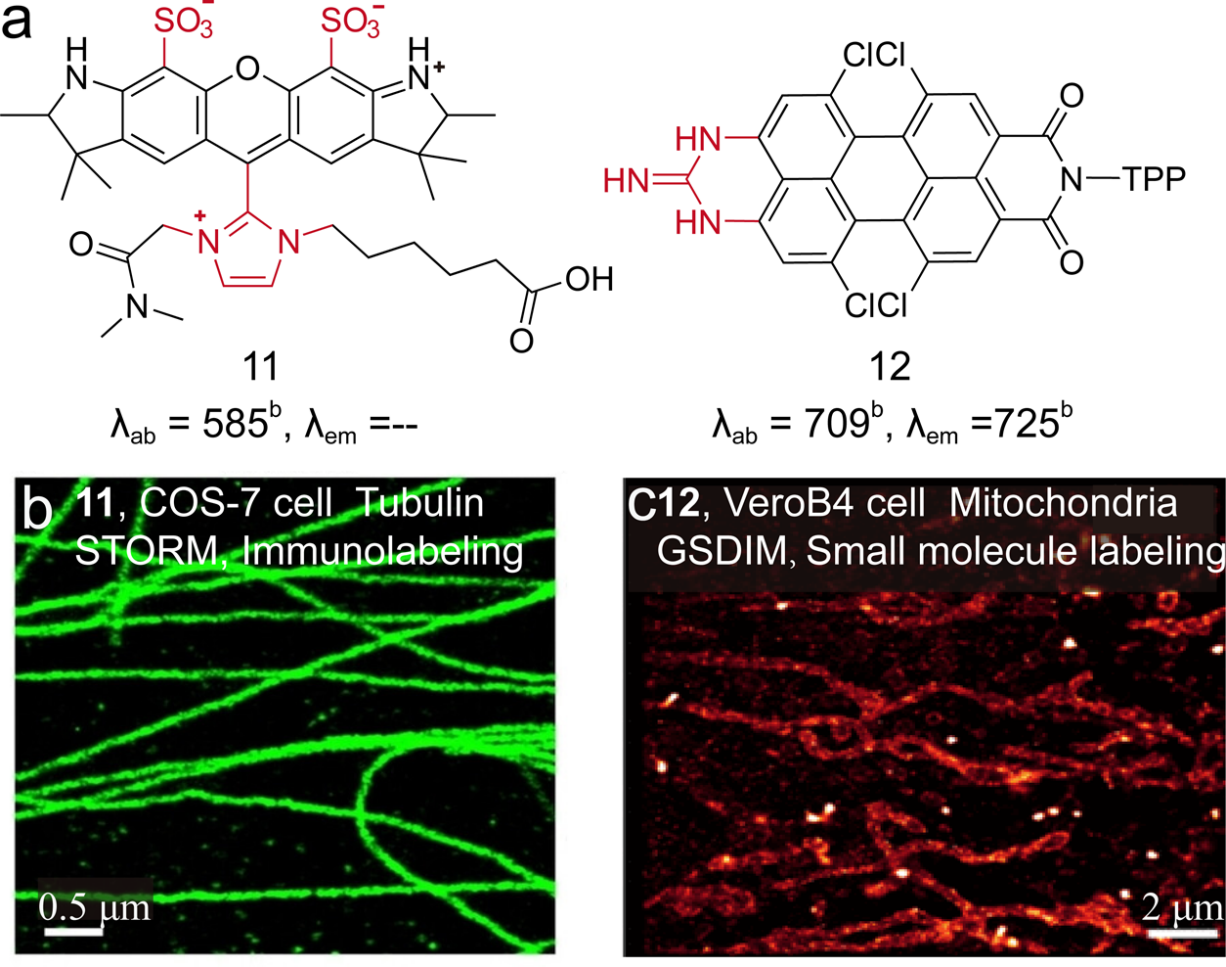
(a) Engineering structures for optimizing switching physics. (b) A typical (d) STORM image of **11**-labeled microtubules in fixed COS-7 cells. Reproduced from ref 61. Copyright 2022, Wiley-VCH. (c) Super-resolved images (right) of mitochondria in fixed VeroB4 cells stained with **12**. Reproduced from ref 62. Copyright 2016, American Chemical Society.

Dyes with photochromic characteristics (Figure 14) are favorable for single-molecule localization imaging because of their intrinsic switching behavior. The photochromic single-molecule process of rhodamines (Figure 14, **13**) was pioneered by the Hell group.^34^ Later modulation of spirolactam substitutions shifts the activation laser to the visible region by the Moerner group (Figure 14, **14**).^64^ Recently, Lavis et al. developed a new photochromic dye^65^ by linking a rhodamine scaffold to a 7-aminocoumarin moiety (Figure 14, **15**). The coumarin unit acts as a photoswitching modulator, enabling both photochromic and spontaneous ring-opening-induced blinking for localization imaging.

**Figure 14:**
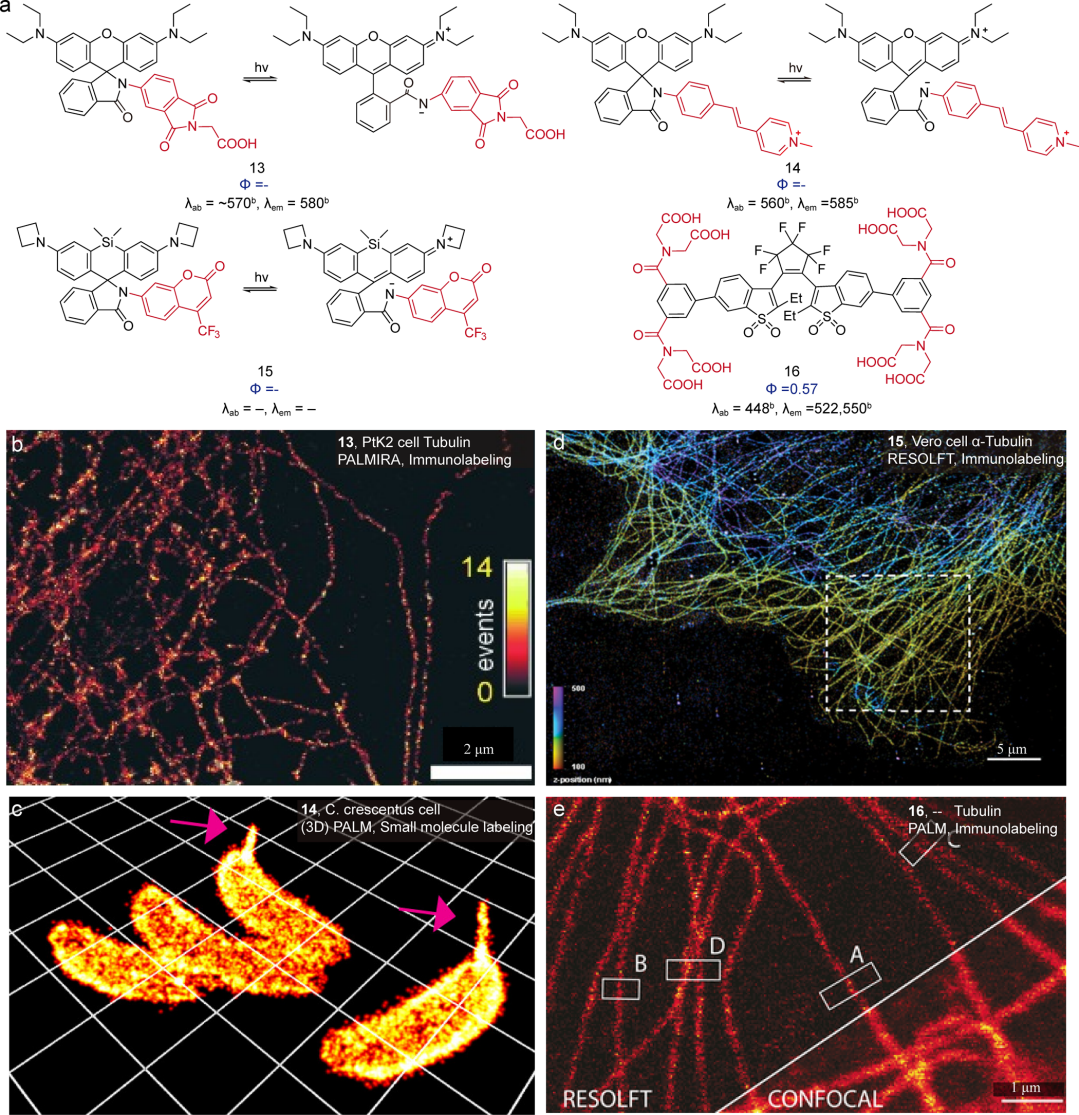
(a) Photochromic dyes. (b) Reconstructed images of the tubulin network in PtK2 cells stained with **13**. Reproduced from ref 34. Copyright 2007, Wiley-VCH. (c) Three-dimensional reconstructed images of six C. crescentus cells. Reproduced from ref 64. Copyright 2014, American Chemical Society. (d) 3D super-resolution image of microtubules stained with an antibody conjugate prepared from NHS ester **15**. Reproduced from ref 65. Copyright 2024, bioRxiv. (e) RESOLFT images of whole fixed Vero cells immunostained with primary antibodies against α-tubulin and **16** attached to the secondary antibodies. Reproduced from ref 66. Copyright 2016, Wiley-VCH.

After successful implementation of rhodamine spirolactams, photoswitchable diarylethene dyes were studied for their single-molecule fluorescence. Hell et al. developed a photoswitchable carboxylated diarylethene dye by introducing excessive carboxyl groups (Figure 14, **16**),^66^ both of which inhibited aggregation and provided switching in aqueous conditions. Wöll and colleagues synthesized naphthyl-substituted derivatives,^67^ extending the emission to 558 nm with an exceptionally low spontaneous ring-closing rate. Subsequently, their studies further systematically investigated the photoswitching of diarylethenes.^68^ Collectively, these efforts positioned diarylethenes as a universal photoswitching platform, where atom-level substituent engineering drives a paradigm shift in the rational design of super-resolution imaging dyes.

Photoreaction is an effective strategy for imparting switching-like behaviors to dyes. Although most of these reactions are nonreversibly performed and the generated signals appear only once, it is possible to localize most dye molecules by controlling the reaction rate. It is possible to directly engineer dark states in advance to obtain sparse activation of these dark dyes during imaging. Switching could be achieved through prior reduction (such as NaBH_4_ ^69^) to break the conjugation system (Figure 15). Thus, oxidation results in the recovery of the dye and generates a single-molecule signal. Schmeranzer group screened the NaBH_4_ reduction strategy for 39 commercial dyes and resolved the ultrastructure of 40 nm synaptic vesicles in brain slices.^70^ Shim and colleagues extended this strategy to DNA-PAINT imaging by reducing Cy3B dyes to a dark state using NaBH_4_,^71^ followed by selective reactivation with 405 nm total internal reflection illumination. This approach keeps unbound probes in a nonfluorescent state, effectively suppressing the background noise. The dual-mode control of the switching kinetics increased the imaging speed by 2 orders of magnitude. Together, these studies establish “reductive caging” as a general strategy for modulating dye switching kinetics, centered on atom-level photophysical control via targeted modifications of the dye core scaffold.

**Figure 15:**
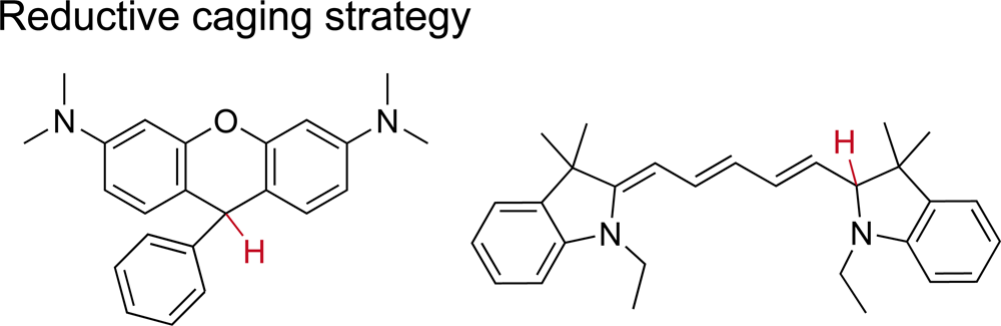
Model structures of reductive caged dyes after treatment with NaBH_4_.

Following the idea of reductive caging, Collot et al. utilized a photo-oxidative strategy to achieve switching for BODIPY dyes (Figure 16, **17**,)^72^ by introducing a furan moiety. Upon excitation, the dye generates singlet oxygen (^1^O_2_), which selectively oxidizes the furan group, restoring the conjugated system, resulting in up to 93-fold fluorescence enhancement. The system cycles in a cascade manner to provide sufficiently bright dyes for localization. Yang et al. designed a cationic Nile Blue dye^73^ by replacing the 5-amino group with a pyrrolidine ring and introducing a phenyl group (Figure 16, **18**). The dye can be switched to the dark state through photoreduction and subsequently restored to the fluorescent state via oxidation.

**Figure 16:**
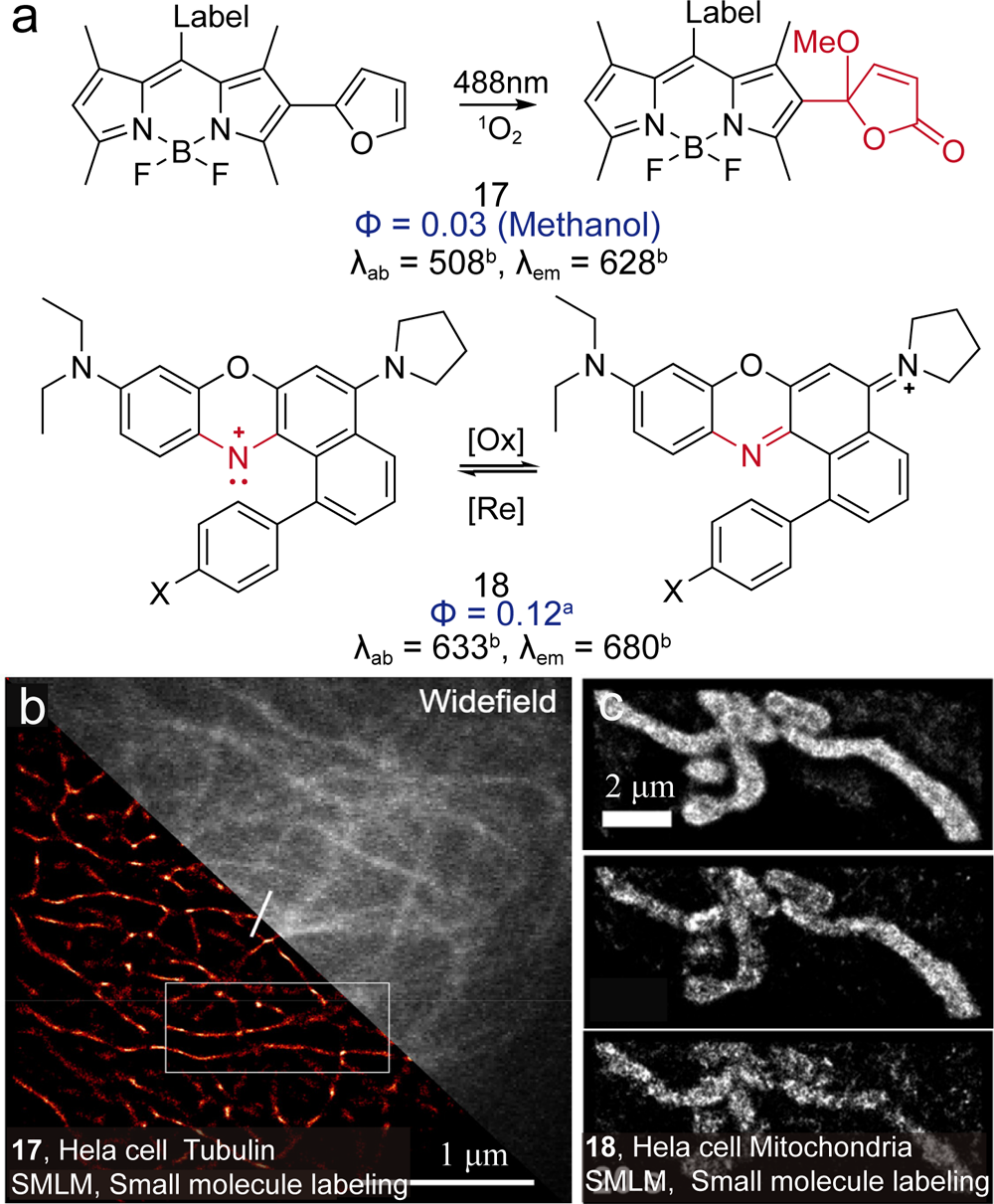
(a) Schematic illustration of the dye switching through photoreaction. (b) Live super-resolution imaging of HeLa cell microtubules. Reproduced from ref 72. Copyright 2024, Wiley-VCH. (c) Live-cell SMLM imaging of mitochondria with **18**. Reproduced from ref 73. Copyright 2024, American Chemical Society.

Other photoreactions can also be utilized to create switchable dyes. Hell group designed and synthesized two fluorescent probes activatable by a combination of β-galactosidase and light.^74^ The photobleaching of the dye could further be utilized for switching. Sauer et al. suggested utilizing the photobluing product Cy3 of Cy5 dye for localization-based super-resolution microscopy.^75^ Almost the same time, Lee et al. utilized the same strategy for single-molecule tracking applications.^76^

A more generalizable photoreaction strategy is caging. Following the “reductive” caging (Figure 17), this strategy forces the dye molecule to exist in its nonconjugate dark form. Under photoactivated light, the dyes restore their fluorescence through caging unit release. The most classic caging unit is the N-acylated nitrobenzoyl caging group. Lavis et al. developed the synthesis of a far-red photoactivatable silicon rhodamine dye (Figure 17, **19**).^77^ The obtained dye is nonfluorescent, and upon 405 nm light activation, its fluorescence is restored with the release of nitroveratryl oxycarbonyl caging groups. We and Yang^78,79^ have developed a nitroso-caged strategy. The developed nitroso-caged rhodamine (Figure 17, **20**) could be activated and excited by the same laser light. Thus, the dye reduces the complexity of the microscopy system by decreasing the number of double excitation lights to one. Xiao and co-workers developed a minimal caging strategy by replacing oxygen atoms in fluorescent dyes with sulfur atoms (Figure 17, **21**).^80^ In addition, to reduce the cytotoxicity concern of photobyproducts, Hell et al. proposed an “intramolecular radical capture” design mechanism.^81,82^ The developed photoactivatable 1-vinylxanthone, i.e., PaX dye (Figure 17, **22**), enabled photoactivation without releasing any byproducts. The PaX dye enables MINFLUX imaging with ultrahigh localization precision and high success rates. Hell group later regulated the radical trap substituents to boost photoactivation efficiency and dye stability (Figure 9, **10**), and established a rational design framework compatible with live-cell imaging.^54^

**Figure 17:**
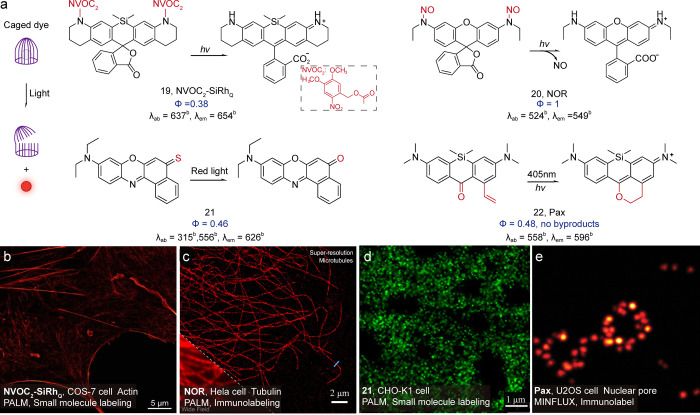
(a) Typical caged dyes. (b) Localization microscopy image (HILO) of COS-7 cells stained with phalloidin conjugate **19**. Reproduced from ref 77. Copyright 2016, Wiley-VCH. (c) Super-resolution image of microtubules immunolabeled with a primary antibody against α-tubulin and a secondary antibody labeled with **20**. Reproduced from ref 78. Copyright 2021, American Chemical Society. (d) PALM reconstruction of **21**-Halo nuclear labeling in a H2B-HaloTag expressing CHO-K1 cells. Reproduced from ref 80. Copyright 2019, American Chemical Society. (e) Live-cell MINFLUX imaging of **22**-labeled NPCs. Reproduced from ref 81. Copyright 2024, Wiley-VCH.

The photoreaction and caging strategies require the utilization of activation laser light. Is it possible to develop dyes with self-driven transitions between dark and bright states? Urano group^83^ ingeniously suggested that the spirocyclization process of photochromic rhodamine spirolactams could be engineered to spontaneously occur at physiological conditions (Figure 18, self-blinking dyes). They suggested that a negative logarithm of the spirocyclization equilibrium constant (p*K*_cycling_ or p*K*_a_, referring to the protonation process of the spirocyclization equilibrium) could be utilized to evaluate the self-blinking possibilities of a rhodamine spirolactam. If a dye has a p*K*_cycling_ value of less than 6, most of its molecules would exist in the dark ring-closed form at physiological 7.4 pH, and the spontaneous transition to the ring-opened bright state would provide single-molecule signals for localization imaging. Thus, the Urano group developed the first self-blinking dye, hydroxymethyl rhodamine, HMSiR (Figure 18, **23**).

**Figure 18:**
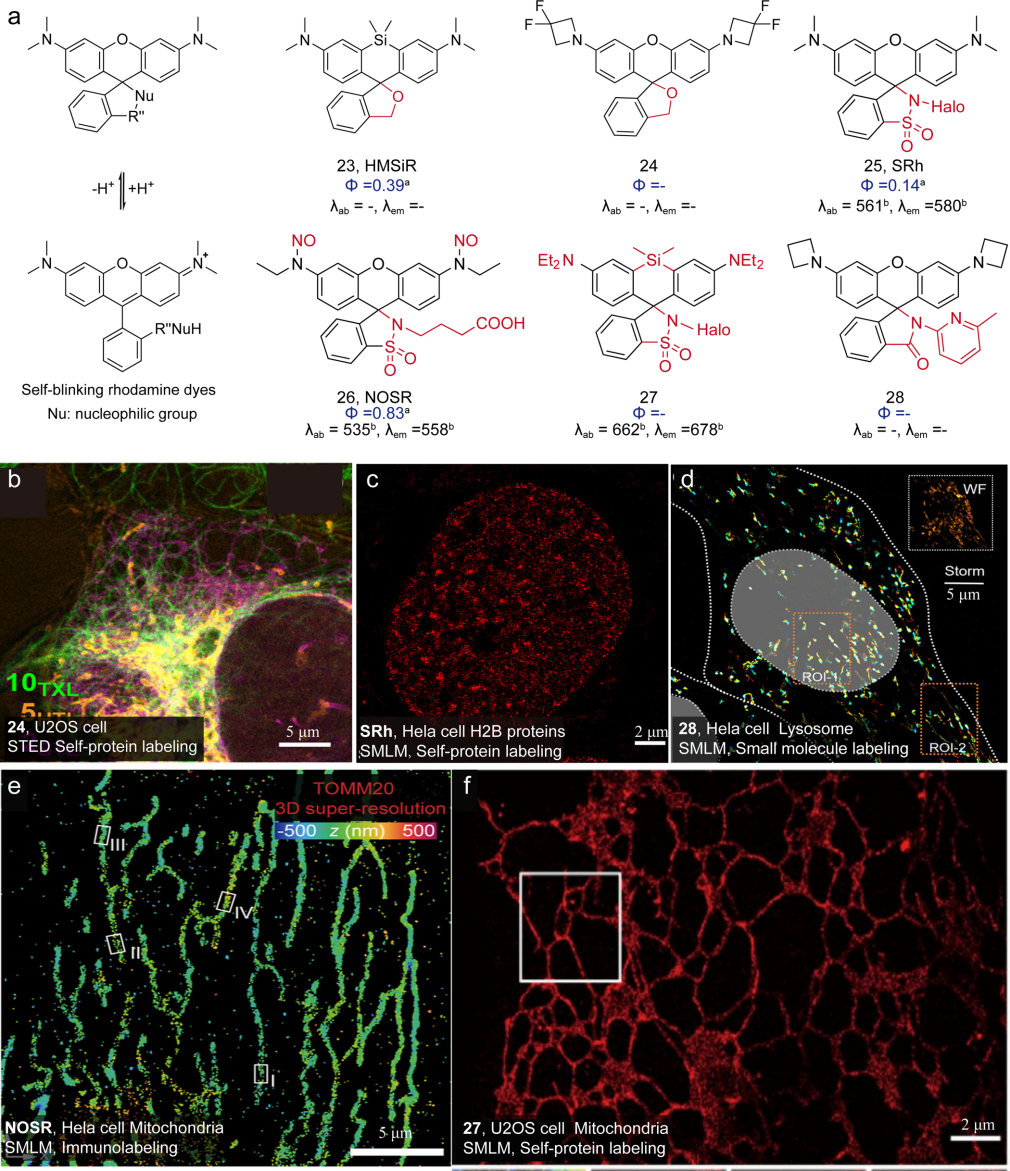
(a) Self-blinking dyes. (b) Three-color live-cell STED image of U2OS cells. Reproduced from ref 35. Copyright 2019, American Chemical Society. (c) Super-resolution reconstruction reveals the distribution of nucleus H2B labeled with **25** using Halo-tag technology. Reproduced from ref 88. Copyright 2023, American Chemical Society. (d) Long-term SMLM imaging of whole-cell lysosomes with **28** in live HeLa cells. Reproduced from ref 97. Copyright 2022, Wiley-VCH. (e) Three-dimensional reconstruction of the mitochondrial outer membrane with **26** in PBS. Reproduced from ref 90. Copyright 2024, American Chemical Society. (f) Reconstruction of endoplasmic reticulum (ER) Sec61β in live U2OS cells through Halo-tag technology with **27**. Reproduced from ref 91. Copyright 2025, American Chemical Society.

Following a similar design, the Schepartz group developed a far-red self-blinking dye, HMSiR 680-Me.^84^ The introduction of a methylamide moiety into the carboxyl group significantly lowered its p*K*_cycling_ to 6.2. The Lavis group also tuned the spirocyclization equilibrium (they suggested another term K_L–Z_, which is the ratio of nonfluorescent lactone and fluorescent zwitterion) through amine exchanges (Figure 18, **24**).^35^ Urano group^85,86^ and Xu^87^ group later independently developed the energetical theory for predicting the p*K*_cycling_ for providing design insights for self-blinking. Although the investigation of the energetic barrier solidly presents the correlation between the structure and spirocyclization equilibrium, it ignores the kinetics of self-blinking, which constitutes the key characteristics for application in molecular localization imaging. We have developed glycine rhodamine (Rh-Gly), which exhibits a p*K*_cycling_ satisfying the “definition” for self-blinking. Yet, reconstruction of this dye failed to depict the labeling structures. This case suggests that the kinetic perspective of self-blinking should not be ignored. Thus, we developed a series of sulfonamide rhodamines (Figure 18, **25**) and investigated their single-molecule fluorescence.^88,89^ In this study, we discovered that the ring-opening kinetics define the temporal resolution of localization imaging and generalize a new term of recruiting rate to define opening kinetics. The recruiting rate is the absolute ring-opening speed, effective for evaluating the temporal resolution of the super-resolution imaging reconstruction for self-blinking dyes (Figure 19).

**Figure 19:**
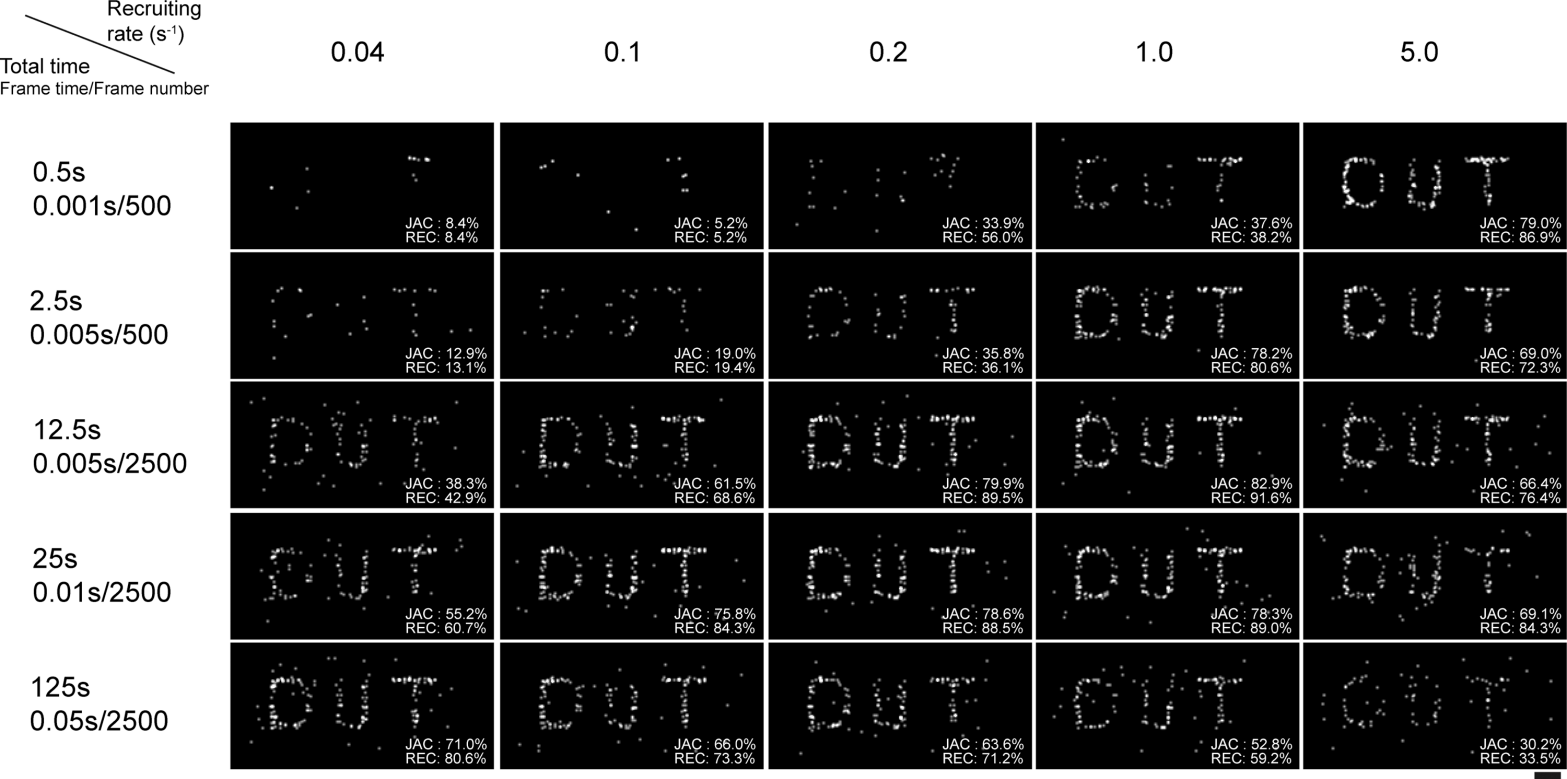
Recruiting rate determines the temporal resolution of self-blinking dyes. Reproduced from ref 88. Copyright 2023, American Chemical Society.

Is there a way to engineer the kinetics of self-blinking? We have implemented a caging strategy for sulfonamide rhodamines (Figure 18, **26**), constructing the first light-triggered self-blinking.^90^ This design successfully transforms the temporal distribution of the single-molecule fluorescence of sulfonamide rhodamines, making these dyes preferable for densely labeled fixed cell structures. Even though this strategy redistributes self-blinking events in terms of temporal resolution, it does not modify the inherent kinetics of self-blinking. Next, we inversed the internal angle of xanthene through heteroatom replacement (Figure 18, **27**), which enlarged the ring-opening energetical barrier and decreased the self-blinking rates.^91^ The developed sulfone amide silicon rhodamine achieved a 30 min-long protein localization reconstruction through one-time labeling without dye exchanging. The Lavis group systematically modulated the electronic properties of the self-blinking dye HMSiR by introducing substituents with varying electronegativities.^92^ Strong electron-withdrawing groups significantly prolong the dye’s off-time and reduce the duty cycle, thereby minimizing signal overlap in single-molecule imaging. Overall, it is noteworthy that the spirocyclization of self-blinking and the photochromic processes present high similarities, as the former provides a protonated form of the photochromic products. We tried to isolate the involvement of thermo-driven spirocyclization and light-activatable photochromic processes for switching amine-acid-based rhodamine spiro amides through single-molecule fluorescence analysis.^42^ It would be of interest if future analysis could be performed to elucidate the full mechanism inside this unique dye.

If one dye molecule could switch, what would happen if they were assembled? Klymchenko et al. investigated ionic pairing between rhodamine B octadecyl ester (R18) and bulky fluorinated tetraphenylborate anions.^93^ By introducing the sterically hindered large anion units, they effectively suppressed π-stacking and self-quenching, allowing nanoparticles (NPs) to retain a quantum yield of 0.20 even at a high dye loading of 5 wt %, with overall brightness reaching six times that of quantum dots. Electrostatic interactions induced by the anion promoted the short-range ordered packing of dyes, enabling ultrafast excitation energy transfer and triggering the collective switching of up to 500 dye molecules. This collective behavior enabled the NPs to function as effective probes for super-resolution imaging.

From the single-dye molecule to the assembly of molecules, the impact factor for switching expands from the natural structure to nearby molecular groups, indicating the significance of the environment in the role of switching. Tachikawa, Majima and colleagues investigated the photoreduction reactions and single-molecule fluorescence properties of BODIPY dyes on TiO_2_ surfaces.^94^ Toomre group discovered that the self-blinking dye HMSiR shows a high environment sensitivity.^95^ The ring-opened state of this dye has a “taste” for the lipid surface. By precisely designing the exchangeable labeling strategy, the same dye enabled over six times delayed photobleaching in aqueous conditions, enabling super-resolution imaging for up to 30 min. We have constructed a styryl-extended BODIPY dye, BDP-Mem, incorporated with quaternary piperazine units.^96^ The hydrophobicity of the dye scaffold provides affinity for hydrophobic membranes, while the positive charge on the quaternary piperazine unit enables retention. The dye exhibits a stable bright state in the membrane’s hydrophobic environment, and its styryl structure provides *cis*–*trans* possibilities for switching. Thus, the utilization of the dye extends to both super-resolution reconstruction of membrane structures in a dSTORM-type manner and single-molecule tracking of the diffusional heterogeneity on the membranes. Later, Xu et al. developed an acid-resistant rhodamine spirolactam photoswitchable dye using an intramolecular hydrogen-bonding strategy (Figure 18, **28**).^97^ By introduction of an amino group at the 3-position of the carboxyphenyl ring, the closed spirolactam structure was stabilized through the formation of NH–O hydrogen bonds, effectively suppressing acid-induced ring-opening reactions for lysosomal imaging.

It is apparent that most previous studies constructed dark-oriented dyes, and they focused on developing activating strategies (photoactivation, e.g., caged dyes or self/environmental-triggered blinking, e.g., self-blinking, environmentally sensitive dyes) due to the facility for switching modulation through external activation rate control. Switching after photoactivation is largely obscured. The switching kinetics (*k*_r_, *k*_d_, and *k*_b_) alongside the activating rate (*k*_a_ and *k*_rc_) produce concrete blinking behaviors for SMLM dyes (or activated dyes). Through our recently reported switching kinetics, it is possible to uncover the structural dependency of the switching efficiency, ε_switch_, for a series of sulfone amide rhodamines. With the enhancement of electron donor capability for the amine groups, those rhodamines exhibit an enhanced switching efficiency with lower molecules existing in bright states, recommending high-density imaging applications (Figure 20). Although we have attempted to summarize the switching kinetics for more dyes, we find that it is not possible, as few have reported this value. Thus, we strongly suggest that studies involving switchable dyes should report these kinetics (*k*_r_, *k*_d_, and *k*_b_) for imaging specialists to select the best dyes.

**Figure 20:**
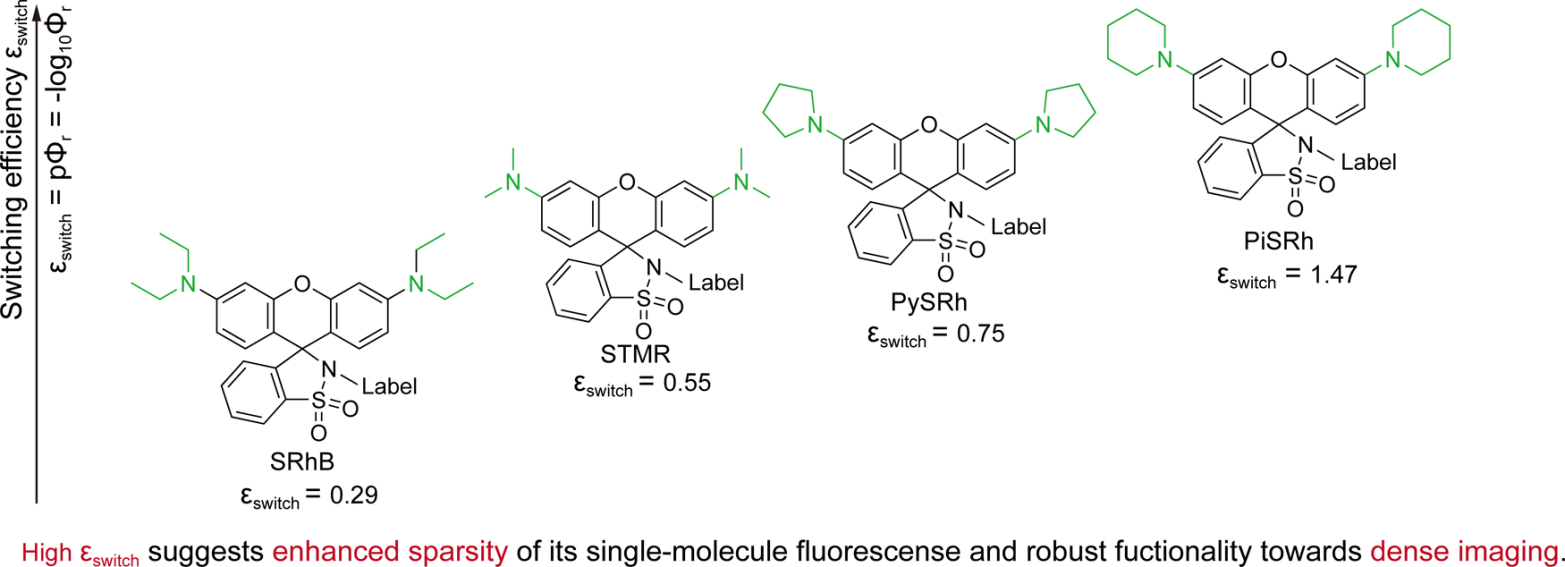
Structural dependency of switching efficiency, ε_switch_, toward amine substitutions for sulfone amide rhodamines.

## Conclusions

Dyes articulate their existence with colors. The evolution of super-resolution microscopy has opened up a new chemical space for century-old dyes. The resolution revolution pushed the boundary of photophysical requirements from the ensemble to a single molecule, producing space for researchers in the dye field to plot. In this review, we summarize the extraordinary expedition in the dye field for molecular localization super-resolution imaging, especially for structural insights into single-molecule fluorescence. The success of single-molecule fluorescence is clarified in two prominent terms: single-molecule brightness and switching kinetics. It is obvious that both terms are strongly influenced by the molecular structure of the interactions between the dye and its surrounding environment. Generally, the structure forms the core building block for brightness and switching kinetics, whereas the interactive environment sets the stage for a single-molecule fluorescence demonstration. For instance, the self-blinking HMSiR not only demonstrates a thermo-driven dark-bright transform but also presents environmental sensitivity. By utilizing the environmental sensitivity, the dye could acquire long-term imaging capabilities. Meanwhile, the single-molecule prosperities, emission rate (photon flux), and dwelling times for different states are highly impacted by the laser power, which hinders future studies. We are optimistic about the proposal of terms like recruiting rate independent of the laser power; more single-molecule prosperity purely reflects the structural impact that would be demonstrated. It is expected that this review will provide insights into the structural aspects of next-generation dye designs.

## Vocabulary

Super-resolution microscopy: Far-field microscopy, surpassing the optical diffraction limit and providing enhanced resolution even at the molecular level.
Point spread function: Diffraction pattern of an ideal point source on microscopy, restricting to the image pattern generated by the statistical photon collection of a single-dye molecule in this review.
Single-molecule fluorescence: Fluorescence acquired from a single-dye molecule, corresponding to the spatiotemporal single-molecule photophysical behaviors (e.g., blinking, photobleaching).
Single-molecule localization: Identification of the molecular spatial position, always referring to acquire the *x*, *y*, and *z* precise location of a dye molecule through fitting its point spread function.
Switching/blinking: Bright–dark cycling (on–off) behavior of fluorescence for a single-dye molecule.
Switching kinetics: Bright–dark (*k*_d_) and dark–bright (*k*_r_) rates, including the kinetics of the activation (*k*_a_) and photobleach (*k*_b_) rates.
